# Predictive value of left atrial appendage lobes on left atrial thrombus or spontaneous echo contrast in patients with non-valvular atrial fibrillation

**DOI:** 10.1186/s12872-018-0889-y

**Published:** 2018-07-31

**Authors:** Fan Wang, Mengyun Zhu, Xiaoyu Wang, Wei Zhang, Yang Su, Yuyan Lu, Xin Pan, Di Gao, Xianling Zhang, Wei Chen, Yawei Xu, Yuxi Sun, Dachun Xu

**Affiliations:** Department of Cardiology, Shanghai Tenth People’s Hospital, Tongji University School of Medicine, NO. 301 Middle Yanchang Road, Shanghai, 200072 China

**Keywords:** Left atrial appendage, Left atrial Thrombus, Left atrial spontaneous echo contrast, Atrial fibrillation

## Abstract

**Background:**

Left atrial appendage morphology has been proved to be an important predictor of left atrial thrombus (LAT) and left atrial spontaneous echo contrast (LASEC) and stroke in patients with non-valvular atrial fibrillation (NVAF). However, the relation between left atrial appendage (LAA) lobes and LAT or LASEC is still unknown. The aim of this study is to investigate the correlation between the number of left atrial appendage lobes and LAT/LASEC in patients with NVAF.

**Methods:**

This monocentric cross-sectional study enrolled 472 consecutive patients with non-valvular atrial fibrillation, who had transthoracic echocardiography (TTE) and transesophageal echocardiography (TEE) prior to cardioversion or left atrial appendage closure (LAAC) from July 2009 to August 2015 in department of cardiology of Shanghai Tenth People’s Hospital. Patients who had significant mitral or aortic valve disease, previous cardiac valvular surgery and other complicated cardiac diseases were excluded. Individuals were divided into two groups:the LAT/LASEC group (16.95%), which comprised patients with LAT or LASEC, as confirmed by TEE; and a negative control group (83.05%).Baseline overall group characterization with demographic, clinical, laboratory data and echocardiographic parameters, alongside with information on medication was obtained for all patients. Subgroup analysis with line chart was applied for exploring the association between LAA lobes and LAT/LAESC. Receptor-operating curves (ROC) were used to test the value of LA anteroposterior diameter detected by different echocardiography methods predicting LAT or LASEC. Multivariable logistic regression analysis was used to investigate independent predictors of LAT/LASEC.

**Results:**

Among 472 patients, 23 (4.87%) had LA/LAA thrombus and 57 (12.1%) had LA spontaneous echo contrast. Compared to the negative group, patients in LAT/LASEC group had higher CHA_2_DS_2_-VASc score (3.79 ± 1.75 vs 2.65 ± 1.76, *p* < 0.001), larger LAD (measured by TTE, 48.1 ± 7.7 vs 44.6 ± 6.5, *P* < 0.001; measured by TEE, 52.2 ± 6.2 vs 46.7 ± 7.1, *P* < 0.001), lower left upper pulmonary venous flow velocity (LUPVFV) (0.54 ± 0.17 m/s vs 0.67 ± 0.26 m/s, CI 95% 0.05–0.22, *P* = 0.003), more left atrial appendage lobes (1.67 ± 0.77 vs 1.25 ± 0.50, *p* < 0.001). There was a good discriminative capacity for LAD detected by TTE (area under the curve (AUC), 0.67, CI 95% 0.61–0.73, *p* < 0.001) and LAD detected by TEE (AUC, 0.73, CI 95% 0.67–0.79, *p* < 0.001). The subgroup analysis based on gender and different LAA lobes yielded similar results (male group: *p* < 0.001;female group: *p* = 0.004) that the number of LAA lobes were significantly associated with LA thrombus or SEC. In multivariable logistic regression analysis, both the number of LAA lobes (odds ratio: 2.37; CI 95% 1.37–4.09; *p* = 0.002) and the persistent AF (odds ratio: 3.57; CI 95% 1.68–7.57; *p* = 0.001) provided independent and incremental predictive value beyond CHA_2_DS_2_-VASc score.

**Conclusion:**

The number of LAA lobes is an independent risk factor and has a moderate predictive value for LAT/LASEC among NVAF patients in China.

## Background

Atrial fibrillation (AF) is one of the most commonly observed arrhythmias in clinical practice with an incidence of 0.77% in China and approximately 1.5–2.0% in the developed world. This arrhythmia is associated with a five-fold risk of stroke, and 20–30% of all strokes are due to AF, thus a higher mortality compared with those without AF [1–3]. Actually, over 90% of embolic stroke was caused by thrombi that originating from left atrial appendage (LAA) [4, 5]. LAA was described as a long, narrow, tubular, wavy, hooked structure with different lobes and a narrow junction and crenellated lumen [6, 7], creating a favorable condition for thrombosis, especially under the situation of AF. Thus it is of great importance to identify the thrombi or signs indicating thrombi formation in LAA. Presence of thrombus, spontaneous echo contrast (SEC) in LA/LAA, or decreased LAA emptying velocity has been reported as markers of thromboembolic risk in non-valvular atrial fibrillation (NVAF) [8–10]. Thrombus was defined as a hyperechogenic non-muscular and non-endocardial mass detected by more than one plane axis during transesophageal echocardiography (TEE), and SEC was defined as smoke-like material with a characteristic swirling motion that persisted throughout the cardiac cycle [11, 12].Actually, the severity of LASEC quantified by different semi-quantitative assessments has been proven to be associated with stroke events in patients with NVAF. It’s reported that denser LASEC was accompanied by a higher risk of LAA thrombus formation in patients with NVAF [13–15].However, in our study, we don’t pay attention to semi-quantitative methods of LASEC which are largely influenced by the experience of the operator. We look LAA thrombus as the densest LASEC. The presence of at least one of them was designated left atrial abnormality.

Remarkably, it is observed that even in patients with AF, the incidence of AF associated stroke varied widely, ranging from 1 to 20% annually [4]. One possible mechanism behind may be that the incidence of thrombi formation in LAA with different anatomical characteristics, i.e., LAA morphology and number of LAA lobes, varied. Now it is recognized that there were four LAA macroscopic morphologies, including cactus LAA, chicken wing LAA, windsock LAA and cauliflower LAA [5, 16–21]. Several recent studies have demonstrated that different LAA morphologies obtained by Cardiac CT or MRI are closely correlated with LASEC, transient ischaemic attacks (TIA) and strokes in patients with AF [16–21]. However, the relationship between LAA lobes and markers of thromboembolic risk (decreased LAA flow velocity, LASEC, LA thrombus) has not been fully characterized in patients with NVAF. Therefore, the aim of this study is to examine whether the number of left atrial appendage lobes could influence the development of left atrial thrombus (LAT) or left atrial spontaneous echo contrast (LASEC) in patients with NVAF.

## Methods

### Study design

We studied 472 patients with all types of non-vavular AF. Left atrial thrombus and spontaneous echo contrast were analyzed by 2D-TEE and classified into two groups (LAT or LASEC positive group and negative group). Simultaneously, LAA lobes were counted during TEE procedure. Then, univariate analysis was performed using the Student’s t-test and chi-square test. Predictors from univariate analysis were used for obtaining logistic regression models that could determine the relative importance of independent predictors of LAAT and LASEC [22].

### Enrollment

This single-center cross-sectional study enrolled patients undergoing TTE and TEE prior to catheter ablation or left atrial appendage closure (LAAC) during a non-valvular AF episode. A total of 472 consecutive participants (males, 57.4%; mean age, 66.1 ± 10.8 years) who were hospitalized at Department of Cardiology of Shanghai Tenth People’s Hospital of Tongji University from July 2009 to August 2015 were referred to our center. AF was identified by an electrocardiogram and met the diagnostic criteria used in 2011 ACCF/AHA/HRS Guidelines for the management of patients with AF [1]. Exclusion criteria included: [i] moderate or severe mitral stenosis; [ii] severe mitral regurgitation; [iii] severe aortic stenosis; [iv] prosthetic mitral or aortic valves; [v] patients with unsuitable images for accurate assessment of TEE surrogate markers of stroke; [vi] congenital heart disease (i.e. atrial septal defect, ventricular septal defect, et al...); [vii] any contraindication to TEE (i.e. esophageal obstruction, esophageal varices, et al); [vii] poor image quality. Then all of these patients were stratified into two subgroups based on with or without LAT or LASEC.

### Initial data collection

All individuals were subjected to thorough history taking and full clinical evaluation. Patient gender, age, heart rate, systolic blood pressure (SBP), diastolic blood pressure (DBP), type of AF, duration of AF, CHA_2_DS_2_-VASc score, antiplateletdrugs or anticoagulant drugs, antiarrhythmic drugs, as well as history of congestive heart failure, hypertension, diabetes mellitus, previous stroke, vascular disease and other related diseases, were recorded. CHA_2_DS_2_-VASc score were calculated with 1 point assigned for a history of congestive heart failure, hypertension, 74 years ≥ age ≥ 65 years, female, diabetes mellitus and vascular disease and 2 points assigned for age ≥ 75 years, a history of stroke or transient ischemic attack (TIA), the maximum score is 9. Specifically, previous stroke also included lacunar infarction.

### Echocardiographic data

Doppler echocardiography was performed using commercially available equipment (Vivid 9 system, General Electric, Horten, Norway) and a variable frequency phased-array transducer. Complete M-mode, two-dimensional, spectral-and color-Doppler images were used to obtain the following measurements: left atrial diameter (LAD), left ventricular end systolic diameter (LVESD), left ventricular end diastolic diameter (LVEDD), interventricular septal thickness (IVST), left ventricular posterior wall thickness (LVPWT), left ventricular ejection fraction (LVEF), pulmonary artery pressure (PAP). All measurements were taken according to the recommendations of the American Society of Echocardiography. Left ventricular ejection fractions were derived from biplane apical 2 and 4-chamber views using the modified Simpson’s rule algorithm [23, 24].

**Fig. 1:**
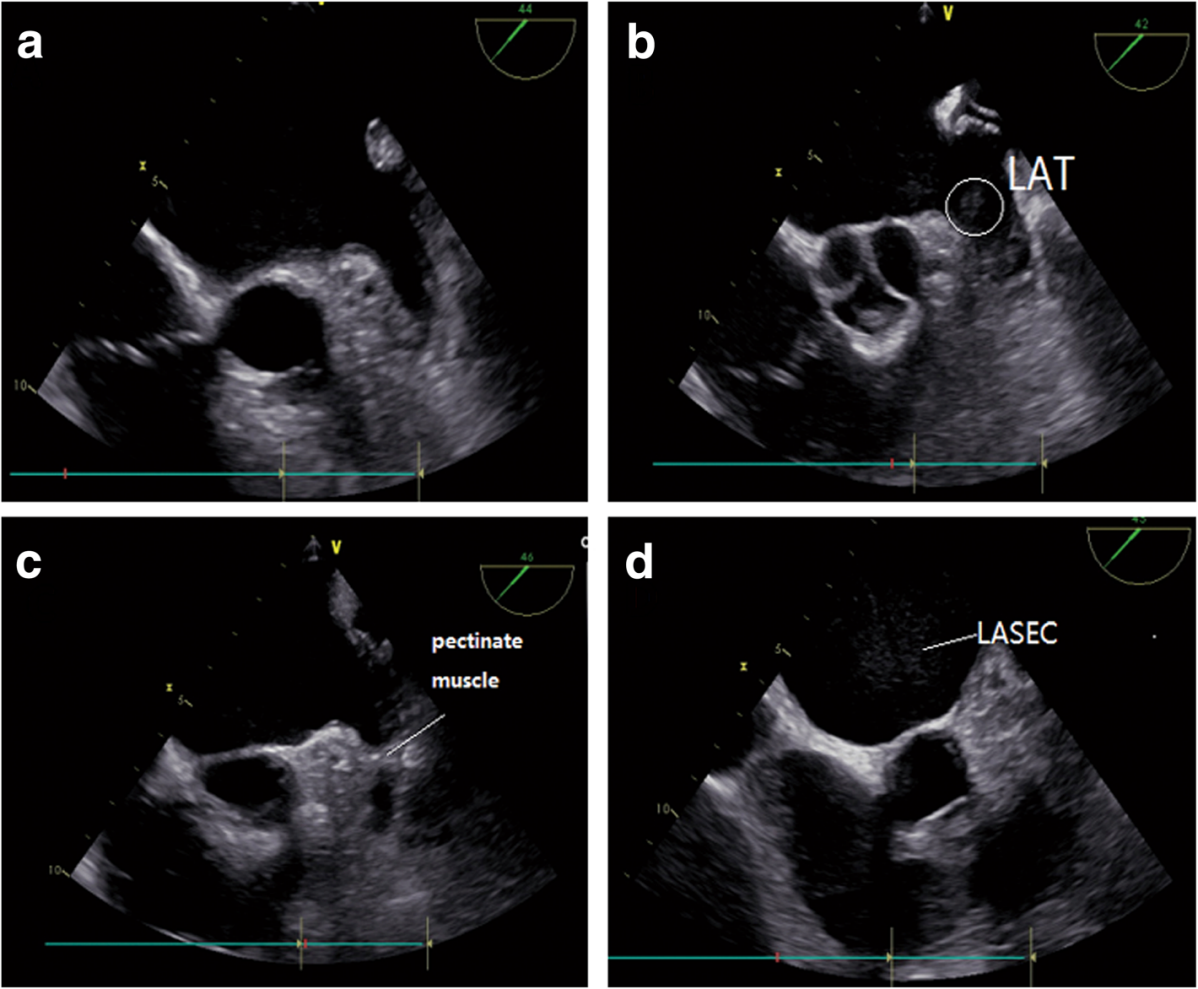
Echo-pattern of LA or LAA thrombosis. The LAA regions are illustrated in a 2D TEE view (45-degree plane). **a** A normal image of left atrium and left atrial appendage (LAA) with one lobe. **b** A mass of thrombus about 9 × 11 mm^2^ in the left atrium near the LAA orifice. **c** A thick pectinate muscle within the LAA cavity, distinct from LAA thrombus. **d** Left atrial spontaneous echo contrast (LASEC) presents as smoke-like swirling echoes

TEE images were acquired with a 6 T phased array multiplane transoesophageal probe (Fig. 1a). LA and LAA were imaged in different tomographic planes from 0° to 180° to detect the presence of thrombusor SEC. LA thrombus was diagnosed by the presence of an echo-dense mass in left atrium or LAA (Fig. 1b), distinct from bulky pectinate muscles [25] (Fig. 1c). Left atrial spontaneous echocardiographic contrast (LASEC) as prethrombotic state having strong correlation with the occurrence of stroke was defined as a pattern of characteristic dynamic smoke-like swirling echoes in LA or LAA (Fig. 1d), distinct from a white noise artifact in the atrial cavity [26]. A pulsed Doppler sample was used to assess the Left atrial appendage flow velocities (LAAFV) [23]. Maximum emptying and filling velocities were estimated from an average of five well-defined emptying and filling waves. Left upper pulmonary venous flow velocity (LUPVFV) was assessed with a pulsed Doppler sample placed 1-2 cm into the left upper pulmonary vein proximal to where it enters the left atrium and at an angle as parallel as possible to the direction of the blood flow from the short-axis view obtained by advancing the TEE transducer to approximately 30 cm from the incisors [27, 28]. In addition, LAD was also obtained by TEE in the 45° plane.

### Classification of left atrial appendage lobes

**Fig. 2:**
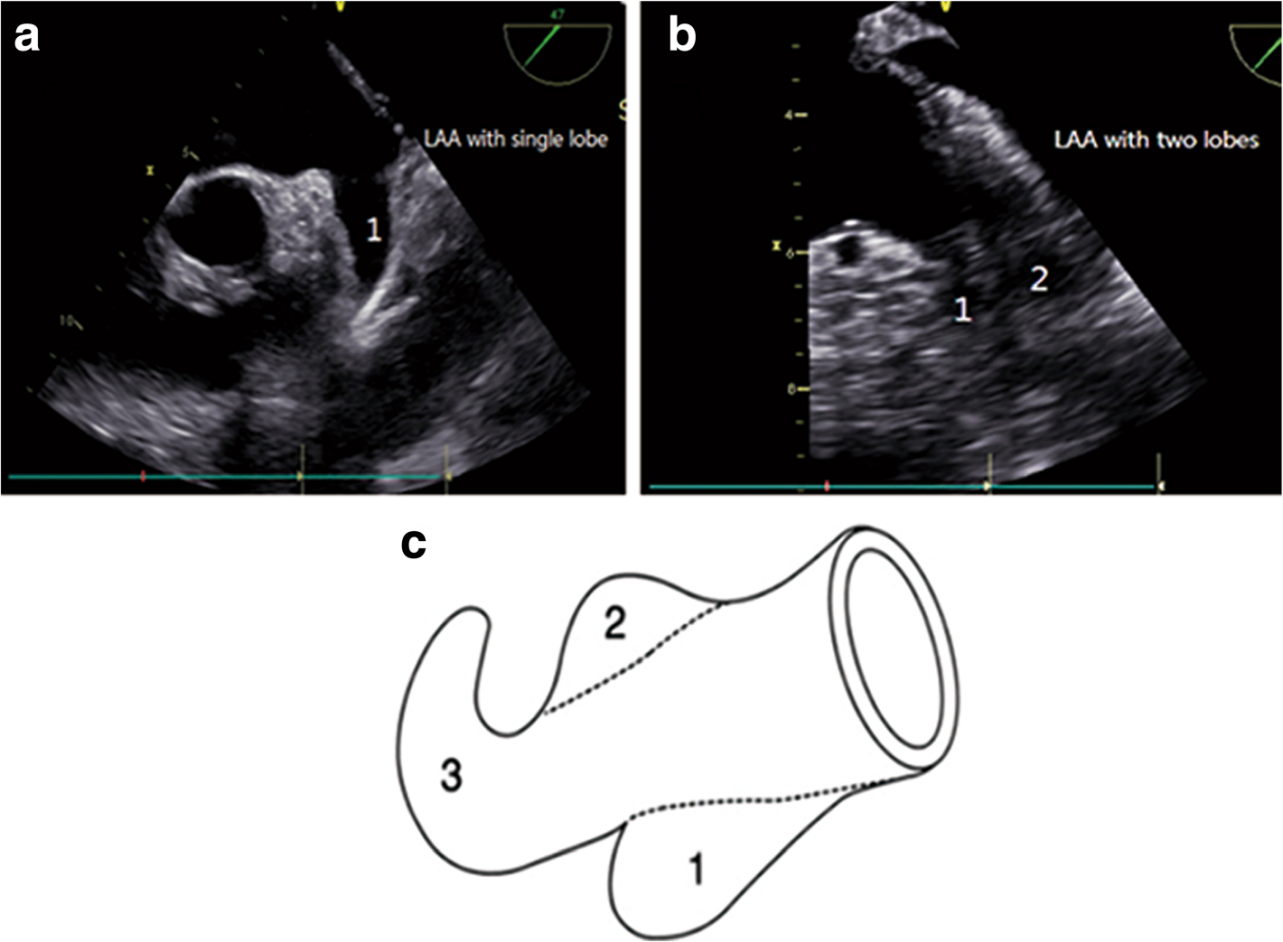
Ultrasound images of LAA with different lobes by TEE. **a** LAA with one wide and deep lobe, composed by a tubular body and a blind-ending sac. **b** LAA with double lobes having shorts shape. **c** Diagram of a left atrial appendage (LAA) shows lobes (1, 2, and 3)

The definition of a lobe includes following criteria: [i] a visible outpouching part demarcated by an external crease from the body of LAA; [ii] the inner diameter was at least 2-mm; [iii] the direction of lobe could be opposite with the main tubular body of LAA; [iv] the anatomic plane was occasionally but not necessarily lain in a same anatomic plane than the main tubular body; [v] the LAA had at least one lobe [29, 30]. Figure 2 showed the morphology of a LAA, the distinct protrusion parts represent lobes. All relevant measurements during TEE and TTE, LAT, LASEC and LAA lobes were confirmed by two experienced cardiologists, who were blinded to the study.

### Statistical analysis

All statistical analyses were performed by SPSS for Windows version 22 (SPSS Inc., Chicago, IL). All continuous data are presented as the mean ± SD and were compared using Student’s t-test and one-way analysis of variance (ANOVA) test for two-level and multiple level grouping variables, respectively. Categorical variables are described as count and percent and were compared using Pearson’s chi-square test (or Fisher’s exact test whenever needed). Then sub-analysis based on gender and different numbers of LAA lobes was used to explore the correlation between LAA lobes and LAT/LASEC, the same as LUPVFV. The receiver operating characteristic (ROC) curve was used to discriminate the power of the LA anteroposterior diameter measured by TTE and TEE in identifying TEE surrogate markers of stroke (LA/LAA thrombus, LASEC) [23]. Comparisons of areas under ROC curves (AUC) were performed between the two measurements, using z-test. Finally, a multivariable logistic regression analysis model was used to identify the significant independent correlates of LAT or LASEC. All potentialco-founders were put into the model on the basis of known clinical relevance or statistically significant association observed in univariate analyses. The odds ratio (OR) and 95% confidence interval (CI) of OR for thrombosis were computed. All tests were 2-sided, and a *P* value < 0.05 was considered statistically significant.

## Results

### Study population

Baseline characteristics of 472 patients were shown in Table 1. LAT or LASEC were found in 80 patients (16.95%, LAT: *n* = 23; LASEC: *n* = 57) by TEE examination. As shown in the table, patients with LAT or LASEC were significantly older but with nearly equal heart rate, systolic blood pressure (SBP), diastolic blood pressure (DBP) and without significant predilection for gender. The percentage distribution of prior congestive heart failure, diabetes, hypertension and previous vascular disease were significantly different between 2 patient groups, more in LAT/LASEC group. Simultaneously, patients with thrombosis had a longer duration of AF and more previous stroke events compared to patients without thrombosis; but these differences were statistically insignificant. In addition, persistent AF during hospitalization was more frequent and CHA_2_DS_2_-VASc score as expected, significantly higher in patients with LAT/LASEC.

**Table 1:** Baseline characteristics of included patients with and without LAT/LASEC

	Total (*n* = 472)	LAT /LASEC group (*n* = 80)	Non-LAT/LASEC group (*n* = 392)	*P* value
Clinical characteristics				
Age(yrs)	66.1 ± 10.8	70.2 ± 9.6	65.3 ± 10.8	< 0.001*
Male (n, %)	271 (57.4%)	40 (50.0%)	231(58.9%)	0.154
HR	86.5 ± 21.8	86.8 ± 18.9	86.4 ± 22.3	0.895
SBP	134.1 ± 18.8	137.0 ± 21.7	133.4 ± 18.1	0.136
DBP	78.7 ± 11.7	79.3 ± 13.1	78.6 ± 11.3	0.642
Persistent AF (n, %)	154 (32.6%)	44 (55.0%)	107 (27.3%)	< 0.001*
Duration of symptoms (months)	45.0 ± 70.4	56.6 ± 76.1	42.4 ± 69.0	0.131
Congestive heart failure (n, %)	73 (15.5%)	24 (30%)	49 (12.5%)	0.003*
Hypertension (n, %)	277 (58.7%)	63 (78.8%)	214 (54.6%)	< 0.001*
Diabetes mellitus (n, %)	77 (16.3%)	20 (25.0%)	57 (14.5%)	0.065
Previous stroke (n, %)	119 (25.2%)	26 (32.5%)	93 (23.7%)	0.174
Vascular disease (n, %)	133 (28.2%)	32 (40.0%)	101 (25.8%)	0.045*
CHA2DS2-VASc score	2.86 ± 1.81	3.79 ± 1.75	2.65 ± 1.76	< 0.001*
Antiplatelet and Anticoagulant drugs				
Aspirin	142 (30.1%)	15 (18.8%)	127 (32.4%)	0.033*
Warfarin	187 (39.6%)	37 (46.3%)	150 (38.3%)	
Dabigatran	47 (10.0%)	13 (32.5%)	34 (8.7%)	
Rivaroxaban	2 (0.42%)	0	2 (0.51%)	

Values are mean ± standard deviation, or number (%)

*LAT* left atrial thrombus, *LASEC* left atrial appendage spontaneous echo contrast, *AF* atrial fibrillation, *HR* heart rate, *SBP* systolic blood pressure, *DBP* diastolic blood pressure. *P < 0.05

### Laboratory examinations and echocardiographic characteristics

These patients’ baseline laboratory examinations and echocardiographic characteristics were shown in Table 2. There were no significant differences between 2 groups in platelets, ALT, AST, serum creatinine, BUN, D-dimer, and it was the same with LVESD, LVEDD, LVPWT, LAAFV and PFO by TTE. However, patients with LAT/LASEC had lower hemoglobin level (130.7 ± 13.1 vs 134.0 ± 18.6, *P* = .049), larger LA anteroposterior diameter measured by TTE (48.1 ± 7.7 vs 44.6 ± 6.5, *P* < .001) or TEE (52.2 ± 6.2 vs 46.7 ± 7.1, P < .001), slightly thicker ventricular septal (10.7 ± 3.0 vs 9.8 ± 1.7, *P* = .018), lower LVEF (60.3 ± 9.3 vs 63.5 ± 8.8, *P* = .005), higher pulmonary artery pressure (30.9 ± 9.8 vs 28.1 ± 8.2, *P* < .011), more LAA lobes (1.67 ± 0.77 vs 1.25 ± 0.50, *P* < .001) and lower LUPVFV (0.54 ± 0.17 m/s vs 0.67 ± 0.26 m/s, *P* < .011). Importantly, both T-test and Chi-Square test showed that LAA lobes of patients with LAT/LASEC were significantly more than other patients (Table 2).

**Table 2:** Laboratory examinations and echocardiographic characteristics of thrombosis group and control group

	Total (*n* = 472)	LAT /LASEC group (*n* = 80)	Non-LAT/LASEC group (*n* = 392)	*P* value
Laboratory examinations				
Hb (g/l)	133.8 ± 17.8	130.7 ± 13.1	134.0 ± 18.6	0.049*
Plt (*10^9/l)	186.9 ± 61.6	183.0 ± 54.3	187.7 ± 63.2	0.576
ALT(U/L)	28.6 ± 61.0	22.6 ± 21.1	30.0 ± 66.7	0.361
AST(U/L)	29.7 ± 75.7	24.1 ± 13.8	30.9 ± 83.9	0.508
sCr (mmol/l)	81.3 ± 25.0	85.7 ± 24.8	80.2 ± 25.0	0.102
BUN (mmol/l)	6.3 ± 2.1	5.7 ± 1.7	6.2 ± 2.1	0.167
D-Dimer	0.53 ± 1.67	0.48 ± 0.43	0.54 ± 1.62	0.700
Transthoracic echocardiographic parameters				
LAD (mm)	45.2 ± 6.8	48.1 ± 7.5	44.6 ± 6.5	< 0.001*
LVESD (mm)	30.6 ± 5.6	31.6 ± 5.3	30.4 ± 5.6	0.092
LVEDD (mm)	47.4 ± 5.0	47.5 ± 5.1	47.3 ± 4.5	0.755
IVsT (mm)	9.96 ± 1.98	10.67 ± 3.00	9.79 ± 1.67	0.018*
LVPWT (mm)	9.58 ± 1.60	10.26 ± 2.44	9.42 ± 1.33	0.418
LVEF (%)	63.0 ± 9.0	60.3 ± 9.3	63.5 ± 8.8	0.005*
Pulmonary artery pressure (mmHg)	28.6 ± 8.5	30.9 ± 9.8	28.1 ± 8.2	0.011*
Transesophageal echocardiographic parameters				
LAD (mm)	47.6 ± 7.3	52.2 ± 6.2	46.7 ± 7.1	< 0.001*
Lobes of Left atrial appendage	1.32 ± 0.58	1.67 ± 0.77	1.25 ± 0.50	<0.001*
1 (n, %)	289 (61.2%)	47 (41.3%)	242 (78.3%)	< 0.001*
2 (n, %)	83 (17.6%)	25 (31.3%)	58 (18.8%)	
> = 3 (n, %)	17 (3.6%)	8 (10.0%)	9 (2.9%)	
LUPVFV (m/s)	0.63 ± 0.24	0.54 ± 0.17	0.67 ± 0.26	0.03*
LAAFV (m/s)	0.35 ± 0.19	0.31 ± 0.13	0.37 ± 0.20	0.217
PFO (n, %)	33 (7.0%)	7 (8.8%)	26 (6.6%)	0.532

Values are mean ± standard deviation, or number (%)

*LAT* left atrial thrombus, *LASEC* left atrial appendage spontaneous echo contrast, *AF* atrial fibrillation, *Hb* hemoglobin, *PLT* platelets, *ALT* glutamate pyruvate transaminase, *AST* glutamicoxaloacetic transaminase, *sCr* serum creatinine, *BUN* blood urea nitrogen, *LAD* left atrial diameter, *LVESD* left ventricular end systolic diameter, *LVEDD* left ventricular end diastolic diameter, *IVST* Interventricular septal thickness, *LVPWT* left ventricular posterior wall thickness, *LVEF* left ventricular ejection fraction, *LAAFV* left atrial appendage flow velocity, *PFO* Patent foramen ovale. *P < 0.05

### LA anteroposterior diameter measurements for identifying LAT/LASEC on TEE

**Fig. 3:**
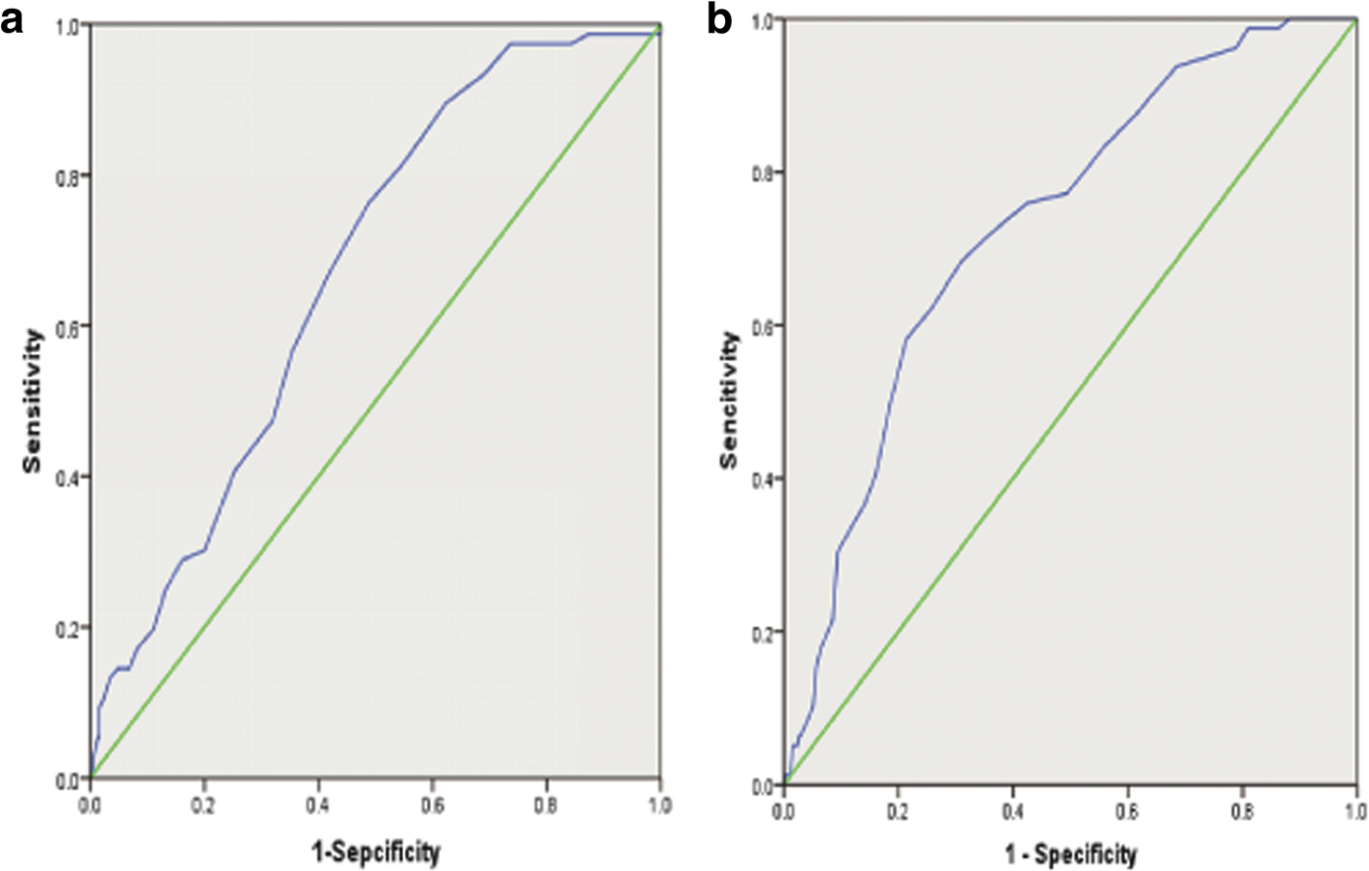
Receiver operating characteristic curve (ROC) of LAD for predicting the presence of LAT/LASEC in NVAF. **a** ROC analysis of LAD measured by TTE for identifying LAT/LASEC; **b** ROC analysis of LAD measured by TEE for identifying LAT/LASEC

ROC analysis demonstrated that a cut-off value of anteroposterior LAD with 44.5 mmby TTEcould predict the presence of LAT/LASEC. At this level, sensitivity was 76.3% and specificity was 51.2%, Area under the curve (AUC) = 0.67 (CI 95% 0.61–0.73, *p* < 0.001). The value of LAD by TEE with a sensitivity of 72.2% and a specificity of 63.6% was 48.5 mm, AUC = 0.73 (CI 95% 0.67–0.79, *p* < 0.001) (Fig. 3 and Table 3). Thus, LAD by TTE and TEE showed a moderately high discriminatory power of the prediction of LAT/LASEC. However, ROC curve comparison for these two measurements revealed anteroposterior LAD by TEE provided greater predicative value than TTE with a significant difference (difference in AUC = 0.06 ± 0.03,Z = 1.99,*P* < 0.05).

**Table 3:** The area under ROC curve of LAD to predict LAT/LASEC

	AUC	SE	*P* value	95% CI
LAD measured by TTE	0.670	0.030	<.001	0.611–0.730
LAD measured by TEE	0.729	0.029	<.001	0.672–0.787

*ROC* receiver operating characteristic, *LAD* left atrial anteroposterior diameter, *LAT* left atrial thrombus, *LASEC* left atrial appendage spontaneous echo contrast, *TTE* transthoracic echocardiography, *TEE* transesophageal echocadiography, *AUC* area under ROC curve, *SE* standard error, *CI* confident interval

### Correlation between the number of LAA lobes and prevalence of LAT/LASEC

**Fig. 4:**
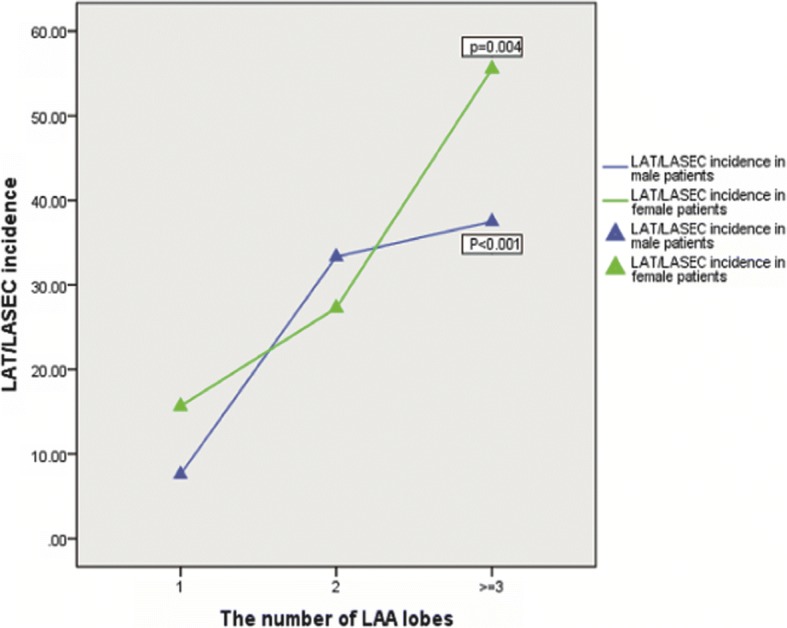
Correlation between left atrial thrombosis and the number of LAA lobes. According to sub-analysis, LAT/LASEC differs significantly among different numbers of LAA lobes in either male (*P* < 0.001) or female (*P* = 0.004) subgroup. Patients with single lobe LAA show a reduced prevalence of thrombus and SEC during TEE compared with patients with multilobe

In our study, distribution of LAA lobes number was from 1 to 4, consistent with previous studies [9]. The most frequent LAA (61.2%) was a single lobe. The number of double and multiple lobes LAA types was 83 (17.6%) and 17 (3.6%). According to ANOVA, patients showed typical differences in LA thrombosis by TEE depending on LAA lobes number: among patients with LAT/LASEC by TEE, the average number of LAA lobes was 1.67 ± 0.77, compared with 1.25 ± 0.50 among patients with non-LAT/LASEC (*P* < 0.001) (Table 2). Moreover, LAT/LASEC incidence rate increased from single lobe to double and further to multiple lobes in sub-analysis in both male (*P* < 0.001) and female (*P* = 0.004) subgroup (Fig. 4).

### The correlation between LUPVFV and prevalence of LAT/LASEC

**Fig. 5:**
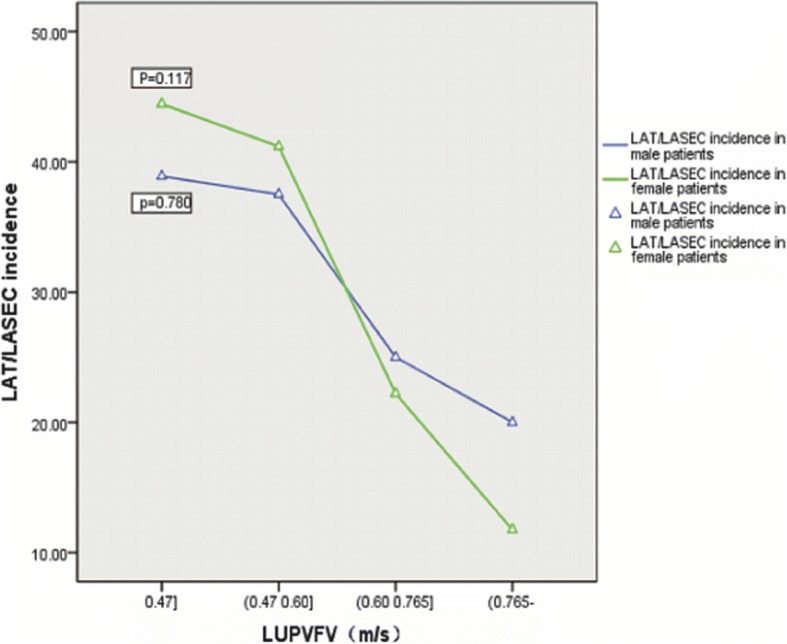
Correlation between left atrial thrombosis and left upper pulmonary venous flow velocity (LUPVFV).The patients in our study who had been detected LUPVFV during TEE were divided into four groups by quartiles. In sub-analysis, LUPVFV decelerated, and the incidence of LAT/LASEC rose gradually from the last quartile to the first quartile, but did not reached statistical significance in both male (*p* = 0.78) and female (*p* = 0.12) subgroup owing to the small sample size

Overall, 95 patients (66 in LAT/LASEC group and 29 in non-LAT/LASEC group) had been detected LUPVFV by TEE. The average LUPVFV was 0.63 ± 0.24 m/s. According to analysis of variance, LUPVFV decreased in patients with LAT/LASEC, compared with non-thrombosis patients (difference in means 0.13 m/s, 95% CI 0.10–0.22, *P* = 0.032) (Table 2). In sub-analysis, these patients were divided into four grades by quartiles. LAT/LASEC incidence rate decreased from first quartile to last quartile, but did not reached statistical significance in both male (*p* = 0.78) and female (*p* = 0.12) subgroup (Fig. 5).

### Independent predictive factors for LAT or LASEC

Multiple candidate clinical predictors and echocardiography measurements were assessed as univariate independent predictors for LAT/LASEC. Our results demonstrated that age, types of AF, LAD on TTE or TEE, antiplatelet or anticoagulation therapy, as well as CHA_2_DS_2_-VASc score were significantly correlated with the presence of LAT/LASEC (Tables 1 and 2). Multivariate linear regression analysis was performed to determine the relative importance of independent predictors of LAT/LASEC, the number of LAA lobes (single, double, multiple, odds ratio 2.37; 95% CI 1.37–4.09; *P* = 0.002), AF types (paroxysmal, persistent, odds ratio 3.57; 95% CI 1.68–7.57; *P* = 0.001) and antiplatelet or anticoagulation therapy (aspirin, oral coagulation medicine, odds ratio 0.36; 95% CI 0.13–0.96; *P* = 0.04) were independent predictors of LAT/LASEC (Table 4).

**Table 4:** Multivariate logistic regression analysis on predictors of LAT or LASEC in patients with NVAF

	B	S.E.	Wald	df	Sig.	Exp(B)	95%C.I.for Exp(B)	
							Lower	Upper
Sex	.430	.377	1.300	1	.254	1.537	.734	3.215
Age	−.012	.022	.316	1	.574	.988	.947	1.031
HR	.008	.009	.811	1	.368	1.008	.991	1.026
SBP	.007	.011	.498	1	.480	1.008	.987	1.029
DBP	−.011	.017	.463	1	.496	.989	.957	1.021
Persistent AF	1.272	.384	10.975	1	.001	3.566	1.681	7.567
CHA2DS2VASc score	.166	.126	1.733	1	.188	1.180	.922	1.511
Anticoagulation therapy	−1.023	.502	4.157	1	.041	.359	.134	.961
LAD by TTE	.046	.039	1.392	1	.238	1.047	.970	1.130
LAD by TEE	.060	.035	2.949	1	.086	1.061	.992	1.136
The number of LAA lobes	.862	.279	9.566	1	.002	2.367	1.371	4.086
Constant	−10.144	2.573	15.544	1	.000	.000		

*LAT* left atrial thrombus, *LASEC* left atrial appendage spontaneous echo contrast, *AF* atrial fibrillation, *HR* heart rate, *SBP* systolic blood pressure, *DBP* diastolic blood pressure, *LAD* left atrial diameter, *TTE* transthoracic echocardiography, *TEE* transesophageal echocadiography, *LAA* left atrial appendage

## Discussion

In the present study, we demonstrated that more LAA lobes number were significantly and independently associated with the presence of LAT and LASEC. Another important finding was that patients with LA thrombosis had a lower LUPVFV. To the best of our knowledge, this is the first study to investigate the roles of LAA lobes number and LUPVFV in predicting left atrial stasis markers: LAT or LASEC.

Accumulative data documented that the presence of thrombus or SEC in LAA/LA are strongly associated with thromboembolism and adverse outcomes in NVAF patients [31–33]. Accordingly, TEE was recommended to evaluate the risk of thromboembolism previous to procedures such as cardioversion, catheter ablation or left atrial appendage closure (LAAC) [1, 34, 35]. In our study, the presence of LAT or LASEC was associated with the risk of thromboembolism assessed by CHA_2_DS_2_-VASc score, due to a higher prevalence of recognized thromboembolic risk factors such as elder, congestive heart failure, vascular disease, hypertension, and diabetes. Furthermore, our results also indicated that LAT or LASEC was closely associated with a larger left atrium, independent of detection methods, such as TTE or TEE, as reported in previous studies [11, 36–38]. However, this association became not significant after adjustment for other potential co-founders. What’s more, our result showed that LAD by TEE provided greater predicted value than by TTE. Although, recent researchers found that LA area in four-chamber view, indexed area-length volumes and diastolic function parameters (E/e’ and e’ velocity) displayed strong correlation with left atrial stasis markers (LAT, LASEC, LAAFV < 20 cm/s) in patients with non-valvular AF [11, 39], our study limited by missing data couldn’t be conducted in these aspects.

Previous studies indicated that the number of lobes was variable, with the prevalence of single lobe ranging from 20 to 42.7%, double lobes from 25.2 to 64.3%, multiple lobes from 26.0 to 35.7% [5, 18, 30]. However, our result showed that patients with a single lobe were in the majority(61.2%), and double lobes 17.9%. These variations may be attributable to the race discrepancy, sample sizes of population and subjective judgment of LAA morphology.

LAA morphology was often classified into four types: (i) Chicken Wing LAA, a main lobe (> 4 cm) with a folded angle under 100°; (ii) Windsock LAA, a main lobe (> 4 cm) with a folded angle over 100°; (iii) Cactus LAA, a main lobe (< 4 cm) with more than two lobes over 1 cm; and (iv) Cauliflower LAA, a main lobe (< 4 cm) with no forked lobes. Such a division of LAA morphology which was originally designed to help practical planning for a transcatheter LAA closure device placement is now widely recognized [40].According to the classification, a Chicken Wing LAA often has only one lobe, sometimes two, while multilobed LAA were more common in NON-Chicken Wing patients. A large number of studies reported an association between LAA morphologies and the risk of TIA and stroke, Chicken Wing LAA had a highest LAA emptying flow velocity and a lowest risk of TIA and stroke [16, 18]. These studies documented that LAA emptying flow and LAAFV decreased in multilobed LAA [18], whereas LAAFV was associated with thrombus formation and stroke, regarded as a predictor of LA thromboembolism [20, 41].^.^Thus, our results support the findings of previous studies: patient with complicated morphology (like non-chicken LAA) had more commonly multilobed LAA, lower LAAFV and was more likely to develop thrombus and SEC than patient with a single lobe LAA. Further research should explore the correlation between LAA lobes and LAAFV.

Additionally, it is generally believed that Chicken Wing LAA is similar to Windsock LAA, and it is also difficult to differentiate Cactus LAA from Cauliflower LAA by morphology. Therefore, such category method is subjective and conflicting. However, the number of LAA lobes by TEE is objective and easy to test, while cardiac CT and MRI are expensive and deleterious, thus we can apply TEE to acquire the number of LAA lobes and examine LAT and LASEC simultaneously.

Additionally, our study documented that left upper pulmonary venous flow velocity (LUPVFV) decreased in LAT/LASEC group. LUPVFV can reflect LA pressure and LV diastolic filling pressure (E/e’), which are important influential factors for LAAFV [27, 42]. Consequently, the combined use of LUPVFV and LAA lobes number can provide additional clinical implications for risk stratification.

### Study limitations

This study has several limitations. First, this study is a single-center study with a relatively small sample. Second, the retrospective design of the study is an additional limitation. Large-scale studies with long-term follow-up are warranted to evaluate the predictive value of LAA lobes for stroke. Third, we investigated a relatively low risk population reflected by a mean CHA_2_DS_2_-VASc score of 2.86. Additionally, the rate of anticoagulation therapy among these patients was high (80.1%), which may affect the rate of LAT and LASEC and thromboembolic events. Thus, our findings could not be adapted to a high-risk AF population.

## Conclusion

More left atrial appendage lobes are associated with significantly higher risk of left atrial thrombus or left atrial spontaneous echo contrast in patients with non-valvular atrial fibrillation. Therefore, the number of LAA lobes is an independent risk factor and has a moderate predictive value for LAT/LASEC among NVAF patients in China.

## Abbreviations

AF: Atrial fibrillation
CT: Computer tomography
LA: Left atrium
LAA: Left atrial appendage
LAAC: Left atrial appendage closure
LAAFV: Left atrial appendage flow velocity
LAD: Left atrial diameter
LASEC: Left atrial spontaneous echo contrast
LAT: Left atrial thrombus
LUPVFV: Left upper pulmonary venous flow velocity
LV: Left ventricle
LVEF: Left ventricular ejection fraction
MRI: Magnetic resonance imaging
NVAF: Non-valvular atrial fibrillation
TEE: Transesophageal echocardiography
TIA: Transient ischemic attack
TTE: Transthoracic echocardiography
